# Biocontrol activity of *Starmerella bacillaris* yeast against blue mold disease on apple fruit and its effect on cider fermentation

**DOI:** 10.1371/journal.pone.0204350

**Published:** 2018-09-21

**Authors:** Chiara Nadai, Wilson José Fernandes Lemos, Francesco Favaron, Alessio Giacomini, Viviana Corich

**Affiliations:** 1 Department of Agronomy Food Natural resources Animals and Environment (DAFNAE), University of Padova, Legnaro, Italy; 2 Interdepartmental Centre for Research in Viticulture and Enology (CIRVE), University of Padova, Conegliano, Italy; 3 Department of Biotechnology, University of Verona, Verona, Italy; 4 Department of Land Environment Agriculture and Forestry (TESAF), University of Padova, Legnaro, Italy; Tulane University Health Sciences Center, UNITED STATES

## Abstract

The reduction of chemical fungicides in agriculture has led to the use of microorganisms as biocontrol agents. *Starmerella bacillaris* is a non-*Saccharomyces* yeast associated with overripe and botrytized grape berries microbiota. Its use has been proposed for wine fermentation because of yeast fructophilic character and high glycerol production. Recently, *S*. *bacillaris* has been demonstrated to possess antifungal activity against *Botrytis cinerea* on the grape. *Penicillium expansum* is the pathogen responsible for the blue mold rot, the most important postharvest disease of apples. These fruits are the raw material of the cider, an alcoholic beverage commonly produced using *S*. *cerevisiae* starter cultures. In this study 14 *S*. *bacillaris* strains have been studied to evaluate their postharvest antifungal activity against *P*. *expansum* on apples. Moreover, the fermentation performances in apple juice of these non-*Saccharomyces* strains were tested, both in single-strain fermentation and in sequential fermentation, together with *S*. *cerevisiae*. Four *S*. *bacillaris* strains, able to significantly decrease blue mold rot symptoms and to increase glycerol content during fermentation have been selected to improve apple and cider quality.

## Introduction

*Penicillium expansum* is the main agent of blue mold rot (also called soft rot) of apple fruit and many other fruit and vegetables during postharvest and causes high economic losses during storage of these commodities worldwide [1]. Blue mold symptoms appear as soft, light brown watery lesions that, at the later stages of decay development, turn blue-green due to formation of conidia [2].

*P*. *expansum* is believed to be the predominant fungal species that produces the mycotoxin patulin in apples and apple products [1]. Patulin is a secondary metabolite that accumulates in fruit leading to serious health problems for consumers [3,4]. Thus, blue mold represents a significant economic problem to both fresh-fruit and fruit-processing industries, since apples and apple products are the main source of patulin in the human diet [5,6], particularly for children that are more exposed and vulnerable than adults [7–9]. In order to protect infants and young children from patulin toxicity, the European Commission regulation [10] established a limit for patulin in apple juice and solid apple products [11].

Traditionally, synthetic chemical fungicides are used to control blue mold incidence in stored fruit [12,13]. However, increased use of these compounds often leads to the establishment of resistant pathogen populations [2,14]. Public concerns for both environment human health have led to regulatory restrictions on fungicides, leading to the search and development of alternative control methods [12,13].

A promising alternative to chemical fungicides strategy is the biological control. Various yeasts, bacteria and filamentous fungi have been identified and characterized for the control of blue mold caused by *P*. *expansum* in fruit and vegetables. Even if the modes of action of these microorganisms have not been fully elucidated, antagonistic yeasts have been selected for their capability to rapidly colonize and grow on surface wounds, thereby competing with the pathogen for nutrients and space [15].

Over the last 20 years, numerous studies on *Penicillium* spp. blue mold biocontrol have been published, but very few products have been patented and registered for commercial use against post-harvest decay of citrus, apples and pears.

Different strains of yeasts, from the genera *Candida*, *Cryptococcus*, *Metschnikowia*, *Kluyveromyces*, *Pichia*, *Rhodotorula*, *Rhodosporidium*, *Saccharomyces* and *Torulaspora* have been studied as biocontrol agents for blue mold [1].

Apple is one of the most important fruit crops in temperate regions worldwide. Apple-based beverages such as cider have been consumed for centuries by the peoples of Eurasia. Alcoholic cider is typically produced in many European countries such as Germany, England, Scotland, France, Spain, Ireland, Slovenia and in North and South America; in recent years, it has experienced the highest growth rates among alcoholic beverages in some European countries [16]. Apple spontaneous fermentation still characterizes the artisanal production but, as in wine making, the cider industries use starter cultures that greatly reduces the risk of spoilage and unpredictable changes in cider flavor, that might yet determine an undesirable loss of characteristic aroma and flavor determinants [17,18]. Therefore, there is a growing interest in isolating and characterizing non-*Saccharomyces* yeasts for development of starter cultures that increase flavor diversity. Additional fruit juices have been studied from a microbiological, compositional and sensory perspective and non-*Saccharomyces* yeasts have been also evaluated mainly for wine and beer production using mixed and sequential inocula with *S*. *cerevisiae* [19] since fruit wines, apple cider and grape wine fermentations share many similarities in microbiological flora and mechanism. Nevertheless, there is limited information on the effect of mixed starter on cider fermentation [20]. Among non-*Saccharomyces* yeasts *Wickerhamomyces anomalus* [20], *Kloeckera apiculata* [21] and *Hanseniaspora valbyensis* [22] have been studied as sequential mixed cultures.

Although several yeasts with antifungal property on apple have been successfully identified, no information is available about the fate of this microorganism during cider fermentation.

During storage, biocontrol protocols include several treatments on apples and, after fruit pressing for fermentation, these yeasts become part of the juice microbiota. Thus, the selection of yeasts with both antifungal and fermentation properties could be of great interest for cider production and at present has been completely unexplored.

*Starmerella bacillaris* (formerly *Candida zemplinina*) is a non-*Saccharomyces* yeast commonly present on grape surface and in enological environments [23]. It has been recently proposed for wine fermentation because of its interesting technological properties: a strong fructophilic character (it preferably consumes fructose than glucose), low acetic acid formation in sweet wines, high glycerol and low ethanol production, ability to enhance wine flavor and mouthfeel [24–29]. During grape must fermentation it has been tested in sequential and mixed yeast inoculations with *Saccharomyces cerevisiae* [27,30,31]. Moreover, Lemos Junior et al. [30] demonstrated the antifungal activity against *Botrytis cinerea* and its potential role as bio-control agent.

In this work, 14 *S*. *bacillaris* strains have been studied to evaluate their post-harvest antifungal activity against *P*. *expansum* on apples. Moreover, the fermentation performances in apple juice of these non-*Saccharomyces* strains were tested, both in single-strain fermentation and in sequential fermentation together with *S*. *cerevisiae* to compare their effect on cider fermentation.

## Materials and methods

### Yeast and *Penicillium expansum* cultures

The yeast strains of *S*. *bacillaris* used in this work, namely PAS13, PAS 55, PAS66, PAS92, PAS103, PAS151, PAS173, FRI719, FRI728, FRI729, FRI751, FRI754, FRI779, FRI7100 [30, 32, 33] were isolated from fermenting must obtained from dried grapes, as described by Lemos Junior et al. [30]. *Saccharomyces cerevisiae* EC1118 (Lallemand Italia, Castel D’Azzano, Italy) was used as control. *P*. *expansum* PVPD2016_3, is a monoconidial preparation isolated from diseased apple fruit. Yeast and mold strains were growth on YPD (Yeast Extract-Peptone-Dextrose, Difco, Milan, Italy) for 24 h and on PDA (Potato-Dextrose-Agar, Difco, Milan, Italy) for 5 days at 25°C, respectively. Occasionally, WL Nutrient Medium (Difco, Milan, Italy) was used to ascertain the absence of contaminant yeasts by colony morphology [34].

### Biocontrol assays

In order to assess the biocontrol activity of the yeast strains against *P*. *expansum*, experiments were performed on apple fruits (cv. Golden Delicious), organically produced, without injuries and with uniform size.

Apples were inoculated according to the method described by Vero et al. [13]. Briefly, after surface disinfection with sodium hypochlorite (0.1% v/v) and rinsing with running tap water, four wounds (5 mm deep × 7 mm wide) were made with a cork borer on the equator of each fruit. Two of the wounds were inoculated with 40 μl of a yeast suspension (10^7^ CFU/ml) and the other two with 40 μl of sterile saline (0.9% NaCl), as a control. For each yeast strain, 2 apple fruits were used (obtaining 4 control and 4 inoculated replicates) in a first preliminary experiment and 7 fruits were used (obtaining 14 control and 14 inoculated replicates) in a second experiment. Inoculated apples were then placed in plastic boxes that were kept at 25 °C. High humidity was maintained by adding some water to the bottom of the trays. After 24 h, the wounds were inoculated with 40 μl of a conidial preparation of *P*. *expansum* (10^4^ conidia/ml). The fruits were then incubated again in the same conditions as above. Approximately 4x10^6^ yeast cells/wound and approximately 4x10^2^ conidia of the pathogen were inoculated. Such pathogen concentration was previously reported to produce infections on 100% of inoculated wounds [13].

After 7 days, the inoculated fruits were examined and the two perpendicular diameters of each lesion radially extending around the wound sites were measured and averaged (LD). For each yeast strain treatment, the disease severity reduction (DSR) was calculated as follow: DSR% = (C − T/C)*100, where C is the average lesion diameter obtained on sites inoculated with *P*. *expansum* (control), and T is the average diameter of lesions obtained on sites inoculated with the yeast and *P*. *expansum* together.

### Colonization of wound site

Growth curves were done in fruit wounds at 25 °C as described by Vero et al. [13]. Wounds (5 mm deep×7 mm wide) were made in surface-disinfected apple fruits with a cork borer.

Each piece of apple (approximately 0.8 g) bearing an inoculated wound was cut and placed in a 15 ml parafilm-capped tube. The wounds were inoculated with 40 μl of yeast suspension of known concentration (10^7^ CFU/ml) and the suspensions were incubated for 11 days (264 h). Control wounds were inoculated with sterile saline (0.9% NaCl). At each sampling time (0, 24, 96, 144, 192 and 264 h), 3 tubes, each one containing a piece of apple per treatment and three controls, were weighted and 7.2 ml of sterile saline was added to them. Samples were then homogenized by vortexing for 2 min. Quantification of viable yeast cells in the resulting mixture was performed by plate count on Malt Agar Medium (Difco, Milan, Italy).

### Fermentation trials

Pre-cultures of each strain used in this work were prepared as described by Bovo et al. [35]. A suitable aliquot of each yeast culture, corresponding to a final concentration of 10^6^ cells/ml was used to inoculate 120 ml-capacity bottles, fitted with closures that enabled the carbon dioxide to escape, containing 100 ml of sterile apple juice (120 g/l of fermenting sugars, pH 3.4) extracted from fresh Golden Delicious apples. In single-strain fermentation, the inoculum concentration was 2–3 x 10^6^ cells/ml. In sequential fermentations, where *S*. *cerevisiae* s EC1118 was added 48 h after the inoculum of *S*. *bacillaris*, the same inoculum size (1–1.5x10^6^ cells/ml) was used for both strains. After yeast inoculation the bottles were incubated at 20°C. All experiments were performed in triplicate. Production of CO_2_ was monitored by weighting the bottles twice a day and calculating the weight loss for each culture. Each fermentation was stopped when the weight loss was lower than 0.1 g after 24. At the end of fermentations, a simple olfactory evaluation was performed by a panel of four trained judges, as described previously [36], focusing on the presence of important defects, such as volatile acidity and sulfur off-flavors.

### HPLC analysis

HPLC analysis was performed to determine the concentration of residual sugars, glycerol, ethanol and acetic acid as described by Nadai et al. [37].

### Statistical analysis

Statistical analysis was performed using the XLSTAT package, vers.2016.02 (Addinsoft, Paris, France). Parametric data were submitted to Student’s t-test or simple analysis of variance (one-way ANOVA) followed by the Tukey’s test [38] as *post hoc* analysis. Non-parametric data were submitted to the Kruskal–Wallis test followed by the Dunn’s test [39] as *post hoc* analysis. Differences were considered statistically significant for *p*-value less than 0.05.

## Results

### Activity of *S*. *bacillaris* strains in reducing blue mold rot on apple

**Fig 1:**
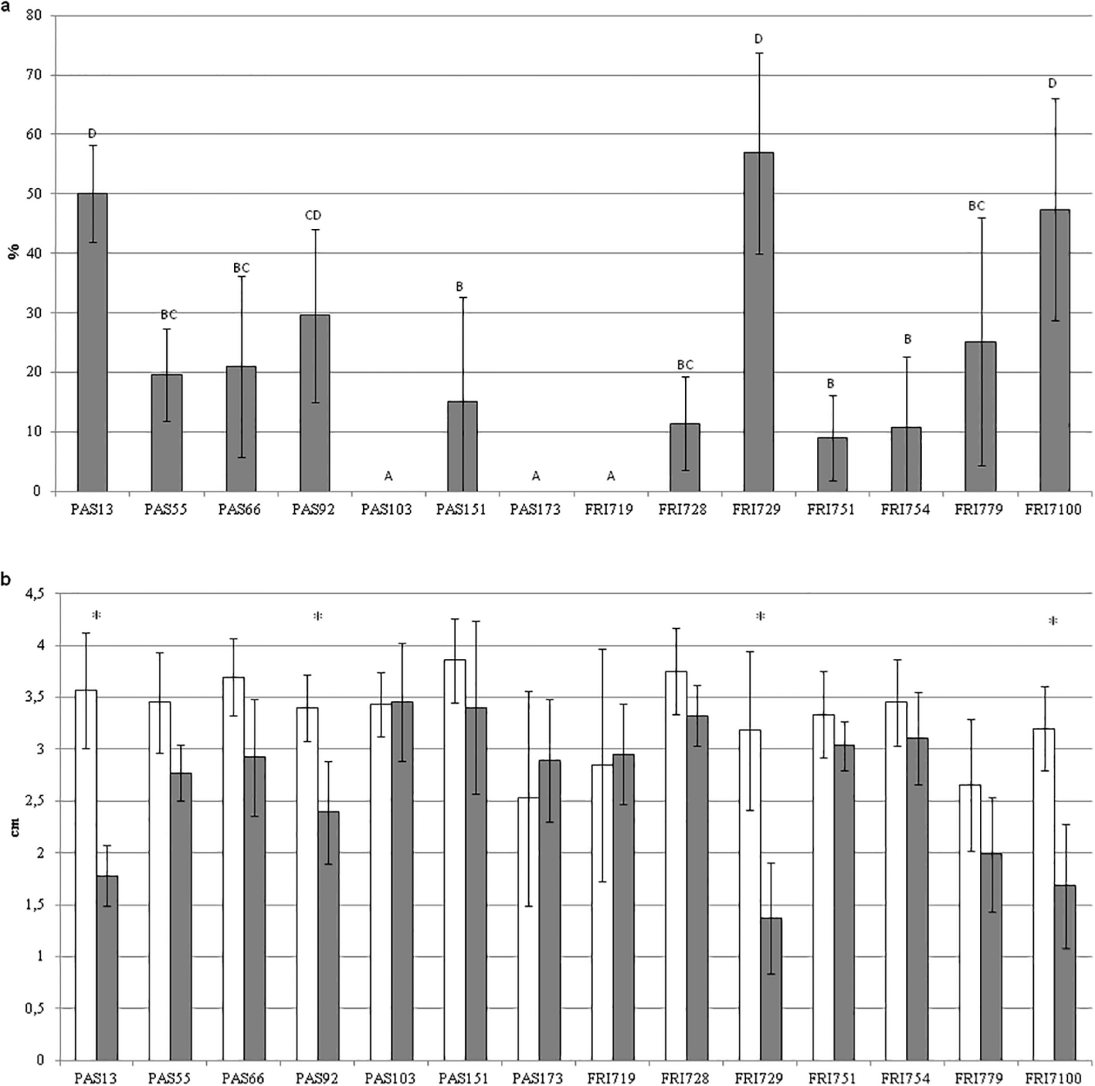
Ability of *S*. *bacillaris* strains to reduce blue mold disease on apples. a) Disease severity reduction (%). Data values with different letters differ significantly according to Dunn’s test (p<0.05). b) Lesion diameters (LD) measured on apple fruits inoculated with *P*. *expansum* (white bars) and on apple fruits treated with *S*. *bacillaris* one day before the inoculation with *P*. *expansum* (grey bars). Asterisks indicate significant differences (*p*<0.05) according to Student’s t-test. Lesion diameters were measured 7 days after *P*. *expansum* inoculation. During the experiment the apples were maintained at 25 °C, at high humidity.

**Fig 2:**
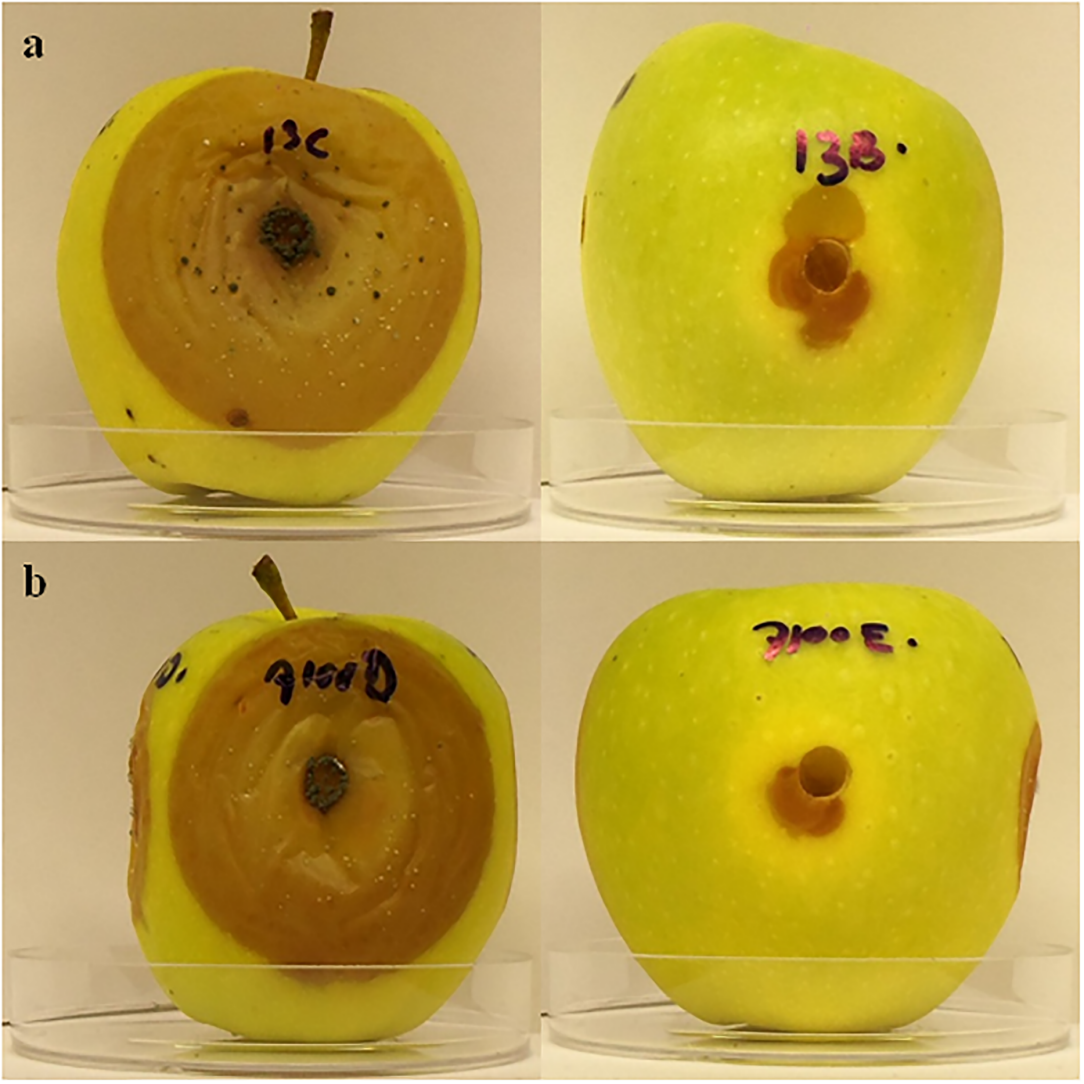
Inhibition of *P*. *expansum* by live cells of *S*. *bacillaris* on Golden Delicious apples. (a) Control on the left, strain PAS13 on the right (b) Control on the left, strain FRI700 on the right. Photographs were taken at day 7 of incubation at 25°C.

A preliminary experiment performed on 14 strains of *S*. *bacillaris* was aimed at identifying the yeast strains with the highest activity in inhibiting apple decay caused by *P*. *expansum*. For each strain, four replicates were performed and two wounds out of four on each apple were used as control (row data are reported in S1 Table). This preliminary screening allowed to select the most active yeast strains to be further investigated (Fig 1). In details (Fig 1a), the disease severity reduction (DSR) value of each strain, compared to the related control, ranged from 0% to 47.4%. Strains PAS103, PAS173, FRI719 had no effect in reducing mycelium growth. Strains FRI7100, FRI729, PAS13 and PAS92 showed the highest and significant (*p* < 0.05) DSR values, 47.4%, 43.6%, 38.5% and 29.6%, respectively (Fig 1a). By comparing the strain lesion diameter with that of the corresponding control, strains FRI7100, FRI729, PAS13 and PAS92 showed significant differences (Fig 1b), resulting the best strains in reducing mycelium growth. In Fig 2 some examples of *S*. *bacillaris* antifungal activity against *P*. *expansum* are reported.

**Fig 3:**
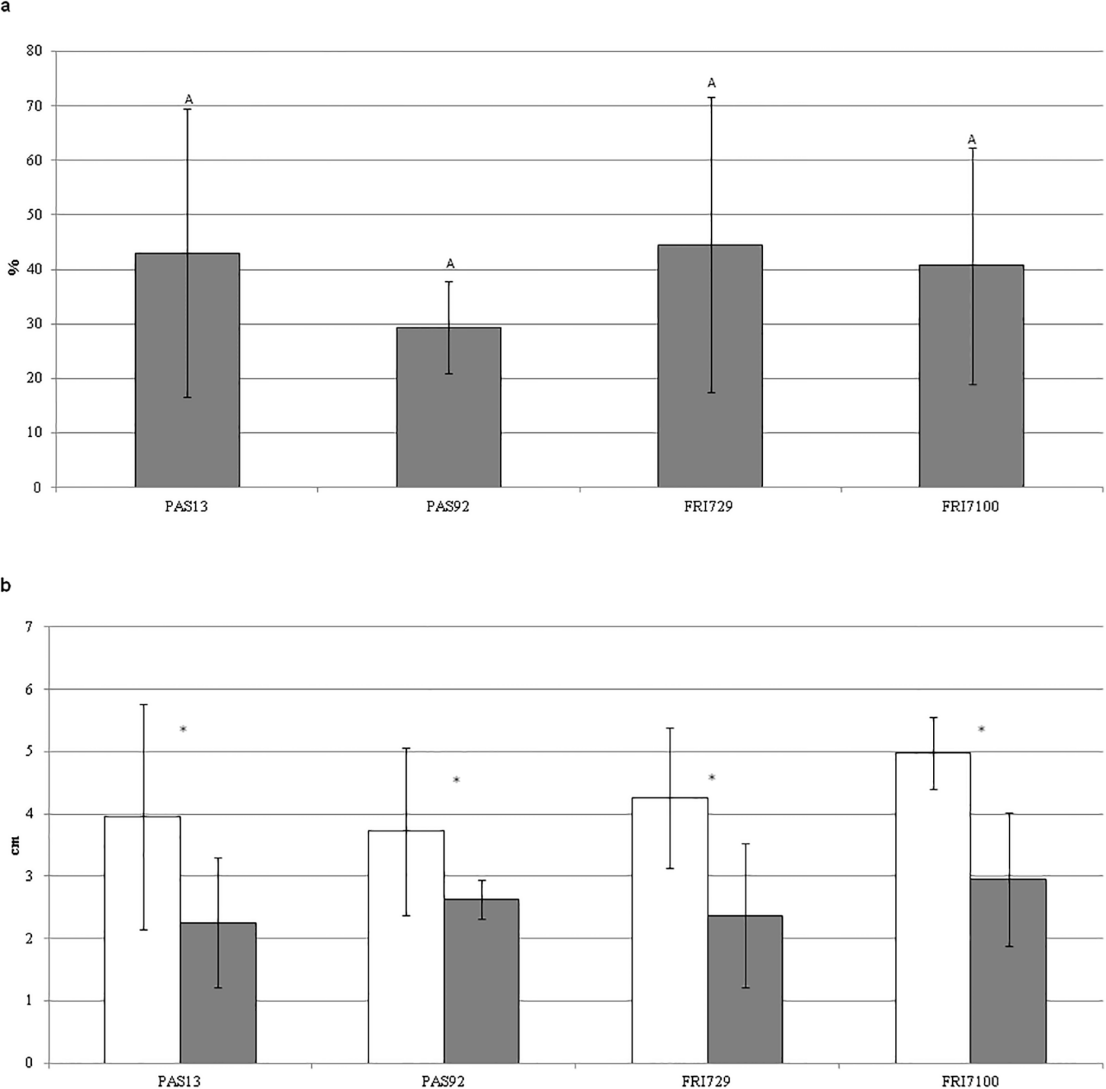
Ability of *S*. *bacillaris* strains PAS13, PAS92, FRI29 and FRI7100 to reduce blue mold disease on apples. a) Disease severity reduction (%). Data values with different letters differ significantly according to Dunn’s test (*p*<0.05). b) Lesion diameters (LD) measured on apple fruits inoculated with *Penicillium expansum* (white bars) and on apple fruit treated with *S*. *bacillaris* one day before the inoculation with *P*. *expansum* (grey bars). Asterisks indicate significant differences (*p*<0.05) according to Student’s t-test. Lesion diameters were measured 7 days after *P*. *expansum* inoculation. During the experiment the apples were maintained at 25°C, at high humidity.

Then, the experiment was repeated with the four most active strains of *S*. *bacillaris* to confirm their activity in reducing *P*. *expansum* lesion size (row data are reported in S2 Table). In this case, each strain was tested on 14 different wounds and its efficacy in reducing *P*. *expansum* symptoms on apples is reported in Fig 3. All four strains were able to induce a significant (*p*<0.05) reduction of lesion diameters with respect to the control. In fact, compared to the corresponding controls, the DSR values (Fig 3a) ranged from 29.4% to 44.5%, in accordance with those obtained in the preliminary experiment.

### Colonization of apple wounds

**Fig 4:**
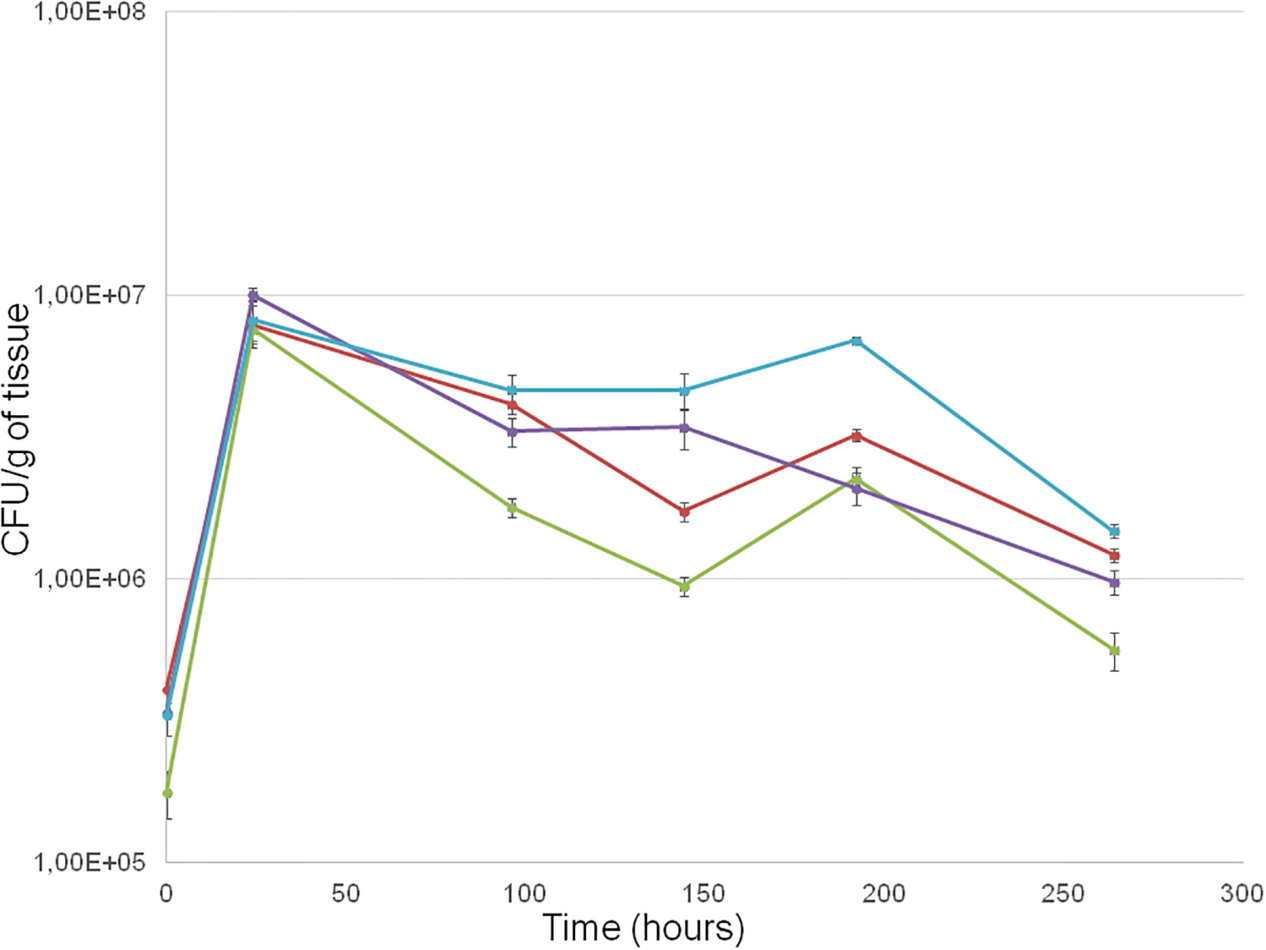
Population dynamics of *S*. *bacillaris* strains PAS13, PAS92, FRI29 and FRI7100 during 264 h of incubation inside apple wounds at 25 °C. ─ FRI729, ─ FRI7100, ─ PAS13, ─ PAS92.

Population dynamics of the 4 selected strains of *S*. *bacillaris* on artificially wounded apples are reported in Fig 4. During the first 24 h, all strains showed similar growth kinetics. Cell concentration, starting with a similar inoculum of about 3.0 x 10^5^ CFU/g of tissue, rapidly increased reaching about 8.1 x 10^6^ CFU/g of tissue. Subsequently, a progressive reduction in the cell number was observed. However, the decline was limited. After 264 h from inoculation, the cell concentration of all strains was higher (on average 1.3 x 10^6^ CFU/g of tissue) than that determined at the beginning of the experiment.

### Fermentation performances in apple juice

Fermentation performances of the 14 strains of *S*. *bacillaris*, both in single-strain and in sequential fermentation with *S*. *cerevisiae* EC1118, were evaluated in apple juice. In sequential fermentations *S*. *cerevisiae* EC1118 was added 48 h after *S*. *bacillaris* strains.

The CO_2_ production was monitored throughout the fermentation process. The fermented juices were analyzed to evaluate glucose and fructose residue and the concentrations of the major fermentation products.

In the single-strain fermentations (Table 1) CO_2_ production after 72 h of incubation (when maximum differences among the strains occurred) was considered in order to evaluate the adaptation ability of the strains to the juice conditions. Moreover, CO_2_ production at the middle and at end of fermentation was also measured.

**Table 1:** CO_2_ production during fermentation, glucose and fructose residues and concentrations of the main fermentation products at the end of single-strain fermentations of apple juice with strains of *S*. *bacillaris*.

Strain	CO_2_/100ml			Glucose (g/l)	Fructose (g/l)	Glycerol (g/l)	Acetic acid (g/l)	Ethanol (%v/v)
	72 h	336 h	672 h					
**PAS13**	0.59±0.01^B^	3.29±0.19^A^	4.91±0.69^A^	16.03±7.82^E^	6.43±0.81^B^	5.43±0.25^A^	1.86±0.19^ABC^	6.07±0.15^A^
**PAS55**	0.56±0.03^B^	3.34±0.10^A^	4.92±0.20^AB^	24.62±0.49^BCD^	9.97±2.24^B^	5.84±0.19^A^	1.85±0.04^A^	5.64±0.22^AB^
**PAS66**	0.52±0.07^B^	2.85±0.34^ABC^	4.58±0.59^AB^	21.97±1.32^DE^	16.80±6.41^AB^	5.58±0.38^A^	1.37±0.05^A^	5.13±0.49^ABC^
**PAS92**	0.47±0.07^BC^	2.89±0.36^ABC^	4.54±0.49^AB^	21.70±2.78^DE^	14.43±7.50^AB^	5.50±0.55^A^	1.42±0.13^ABC^	4.63±0.88^BC^
**PAS103**	0.53±0.01^B^	3.10±0.20^AB^	4.66±0.28^AB^	21.65±1.86^DE^	10.52±3.75^B^	5.57±0.33^A^	1.50±0.13^ABC^	5.42±0.32^ABC^
**PAS151**	0.57±0.14^B^	3.20±0.48^AB^	4.43±0.42^AB^	27.89±2.36^ABCD^	10.01±8.95^B^	5.52±0.43^A^	1.47±0.09^ABC^	5.11±0.52^ABC^
**PAS173**	0.55±0.01^B^	3.04±0.07^ABC^	4.60±0.09^AB^	24.29±0.93^CD^	9.42±2.35^B^	5.30±0.41^AB^	1.46±0.14^ABC^	5.30±0.26^ABC^
**FRI719**	0.12±0.01^C^	2.50±0.12^BC^	3.99±0.14^BC^	32.11±0.26^ABC^	17.40±3.81^AB^	5.23±0.24^AB^	1.16±0.53^C^	5.35±0.28^ABC^
**FRI728**	0.54±0.06^B^	2.68±0.15^ABC^	3.92±0.20^BC^	32.60±0.40^AB^	17.99±3.15^AB^	4.86±0.29^AB^	1.52±0.11^BC^	4.79±0.40^ABC^
**FRI729**	0.45±0.04^BC^	2.73±0.16^ABC^	4.20±0.21^B^	28.63±0.51^ABCD^	15.03±3.03^AB^	5.48±0.04^A^	1.74±0.18^ABC^	5.42±0.18^ABC^
**FRI751**	0.41±0.08^BC^	2.58±0.34^BC^	4.13±0.42^BC^	34.02±0.92^A^	15.79±5.22^AB^	5.95±0.91^A^	1.78±0.20^ABC^	4.13±0.68^C^
**FRI754**	0.44±0.03^BC^	2.83±0.16^ABC^	4.24±0.25^B^	31.02±1.13^ABC^	15.42±2.58^AB^	5.91±0.97^A^	1.35±0.09^AB^	4.12±0.89^C^
**FRI779**	0.74±0.03^A^	2.35±0.10^C^	3.10±0.11^C^	14.25±0.15^E^	27.49±0.61^A^	4.02±0.13^B^	1.72±0.09^ABC^	4.09±0.16^C^
**FRI7100**	0.58±0.07^B^	3.33±0.19^A^	4.94±0.19^AB^	25.53±4.65^BCD^	10.30±2.56^B^	5.92±0.16^A^	1.71±0.25^ABC^	4.26±0.80^BC^

Data are expressed as the average of three replicates ± standard deviations. Different letters indicate significant differences between values according to Tukey’s test (p<0.05).

Fermentation performances were very similar among *S*. *bacillaris* strains. None of the tested strains was able to finish the fermentation after 672 h (Table 1). As expected, *S*. *bacillaris* strains produced limited CO_2_ amounts (3.10–4.94 g/100 ml). Strain FRI719 showed a delay in the fermentation start (0.12 g/100 ml of CO_2_ after 72 h). Strain FRI779 showed the lowest CO_2_ production after 672 h (3.10 g/100 ml of CO_2_).

Sugar consumption confirmed the fructophilic character of *S*. *bacillaris* [25]. In fact, all strains except FRI779 consumed more fructose than glucose (Table 1). Residual sugars were high (from 22.46 to 50.59 g/l) and were related to a limited ethanol production (from 4.09 to 6.07% v/v). The production of secondary metabolites was strain dependent. As expected, glycerol production was generally very high (from 4.86 to 5.95 g/l) except for FRI779 (4.02 g/l). Acetic acid concentrations were very high (from 1.16 to 1.86 g/l) with respect to the average *S*. *cerevisiae* production, as previously reported [40].

To assess strains performances in sequential fermentations (Table 2), the fermentation vigor (i.e. the amount of CO_2_ produced by yeasts after 48 h of incubation) was evaluated together with the CO_2_ production after 360 h (when the EC1118 single-strain fermentation was completed) and at the end of fermentation (528 h).

**Table 2:** CO_2_ production during fermentation, glucose and fructose residues and concentrations of the main fermentation products at the end of sequential fermentations of apple juice with *S*. *bacillaris* strains and *S*. *cerevisiae* EC1118.

Strain	CO_2_/100ml			Glucose (g/l)	Fructose (g/l)	Glycerol (g/l)	Acetic acid (g/l)	Ethanol (%v/v)
	48 h	360 h	528 h					
**PAS13**	0.05±0.01^B^	5.70±0.28^AB^	6.02±0.02^A^	-	2.02±0.15^A^	4.92±0.12^CDE^	0.76±0.11^AB^	8.87±0.07^A^
**PAS55**	0.04±0.02^B^	5.65±0.08^AB^	5.89±0.16^A^	-	2.56±1.02^A^	5.08±0.02^E^	0.65±0.16^AB^	8.70±0.12^A^
**PAS66**	0.05±0.03^B^	5.23±0.55^AB^	5.94±0.22^A^	-	3.78±1.61^A^	4.84±0.15^ABCDE^	0.67±0.15^AB^	8.70±0.06^A^
**PAS92**	0.08±0.01^B^	5.19±0.59^AB^	5.93±0.07^A^	-	3.57±0.68^A^	4.99±0.33^DE^	0.77±0.14^AB^	8.73±0.10^A^
**PAS103**	0.04±0.01^B^	5.45±0.15^AB^	5.92±0.16^A^	-	2.95±1.00^A^	4.70±0.03^ABCD^	0.92±0.11^A^	8.72±0.09^A^
**PAS151**	0.07±0.02^B^	5.49±0.08^AB^	5.92±0.17^A^	-	3.82±1.07^A^	5.09±0.11^E^	0.81±0.05^AB^	8.70±0.09^A^
**PAS173**	0.04±0.02^B^	5.49±0.07^AB^	5.91±0.05^A^	-	3.66±0.68^A^	4.93±0.12^CDE^	0.73±0.01^AB^	8.69±0.05^A^
**FRI719**	0.04±0.00^B^	5.46±0.28^AB^	6.01±0.14^A^	-	4.80±2.83^A^	4.85±0.41^ABCDE^	0.76±0.10^AB^	8.80±0.16^A^
**FRI728**	0.03±0.02^B^	4.97±0.53^B^	5.87±0.23^A^	-	4.14±1.78^A^	4.77±0.08^ABCDE^	0.66±0.14^AB^	8.91±0.13^A^
**FRI729**	0.08±0.02^B^	5.09±0.09^B^	6.04±0.12^A^	-	4.02±1.30^A^	5.11±0.20^DE^	0.80±0.05^AB^	8.84±0.02^A^
**FRI751**	0.03±0.01^B^	5.50±0.14^AB^	6.07±0.08^A^	-	3.52±1.50^A^	4.45±0.05^ABC^	0.65±0.02^AB^	8.91±0.12^A^
**FRI754**	0.04±0.02^B^	5.28±0.04^AB^	6.02±0.04^A^	-	2.42±0.74^A^	4.85±0.11^BCDE^	0.71±0.09^AB^	8.90±0.07^A^
**FRI779**	0.05±0.03^B^	5.05±0.21^B^	6.06±0.05^A^	-	2.77±0.71^A^	3.76±0.23^AB^	0.62±0.15^AB^	8.89±0.07^A^
**FRI7100**	0.05±0.01^B^	5.47±0.18^AB^	5.97±0.10^A^	-	4.01±0.71^A^	4.95±0.10^DE^	0.78±0.02^AB^	8.77±0.07^A^
**EC1118**	1.13±0.09^A^	6.00±0.09^A^	-	-	2.57±0.59^A^	3.22±0.14^A^	0.50±0.09^B^	8.73±0.06^A^

Data are expressed as the average of three replicates ± standard deviations. Different letters indicate significant differences between values according to Tukey’s test (p<0.05).

As expected, fermentation vigor in sequential fermentations was always lower than that of EC1118 in single strain fermentation and no significant differences in CO_2_ production were observed among sequential fermentations containing different *S*. *bacillaris* strains. After 360 h from inoculation, when EC1118 single-strain fermentation was completed, sequential fermentations revealed lower CO_2_ productions compared to EC1118. Hence, sequential fermentations had a lower fermentation rate than that of EC1118 single-strain fermentation.

A very low fructose residue was always present (from 2.02 to 4.80 g/l) in the fermented juice, while glucose was entirely consumed. Ethanol concentration in EC1118 single-strain fermentation (8.73% v/v) was not significantly different than those measured in sequential fermentations with *S*. *bacillaris* strains (ranging from 8.11 to 8.91% v/v). These values were consistent with the initial sugar concentration present in the apple juice. Glycerol concentration in each sequential fermentation was higher than that of EC1118, ranging from 3.76 to 5.11 g/l, whereas EC1118 single-strain fermentation produced only 3.22 g/l of glycerol. In contrast, PAS13, PAS55, PAS92, PAS151, PAS173, FRI729, FRI754 and FRI7100 sequential fermentation showed glycerol levels significantly higher than those found in EC1118 single strain fermentation. Acetic acid concentrations were very limited and lower than the levels found during *S*. *bacillaris* single-strains fermentations (ranging from 0.62 to 0.92 g/l). Strain EC1118 also produced a low acetic acid level (0.50 g/l). No defects after olfactory evaluation of all the fermented products obtained by sequential fermentations were found.

## Discussion

Biological control is now considered one of the best alternatives to the use of synthetic fungicides against fruit postharvest molds, in terms of more sustainable fruit production and higher consumer health benefit [41]. Yeasts have been extensively studied as promising biocontrol agents because of their simple nutritional requirements, the ability to colonize dry surfaces for long periods of time and their rapid growth in bioreactors. Moreover, they do not produce allergenic spores, mycotoxins or antibiotics as many fungi or bacteria do [42]. Regarding fermented fruit, as grape and apple, although several microorganisms with antifungal property have been successfully identified on fruits, few studies are available about the fate of these microorganisms during alcoholic fermentation [30,43].

In this work, 14 *S*. *bacillaris* strains, previously reported to possess antifungal activity against *B*. *cinerea* on grapes [30], have been studied for their potential biocontrol efficacy against blue mold of apples caused by *P*. *expansum*. *S*. *bacillaris* strains are osmotolerant and psychrotolerant (or psychrotrophs). They are also characterized by a fructophilic character, poor ethanol yield and high glycerol production [44,45]. Ecological studies have revealed the presence of this species on grape berry surface and during spontaneous fermentations of musts, i.e. performed without addition of commercial yeasts, in several countries [46–49], suggesting that it has a specific role in the alcoholic fermentation process. It was also found in China on the surface of apples and in apple juice processing plants [50,51].

The antagonistic activity of the strains was tested on wounded apples, artificially inoculated with a *P*. *expansum* strain isolated from apples. Applying 10^5^ yeast cells per wound, 4/14 strains were able to significantly (*p*<0.05) reduce *P*. *expansum* growth and lesions at 25 °C. The disease severity reduction (DSR), was from 29.4% to 44.5%. Comparable values of disease reduction were found in similar assays in which another non-*Saccharomyces* yeast, *Metschnikowia pulcherrima*, was tested [52].

Although previous findings suggested the presence of *S*. *bacillaris* on apple surface [51], the hypothesis that the four identified antagonistic strains could grow on apples and colonize artificial wounds was clearly verified. These studies are yet another example of the aptitude of biocontrol agents to survive and multiply in wounds in competition with pathogens for fruit infection [53,54]. All strains showed a high population concentration after 264 h (around 1.3 x 10^6^ CFU/g of tissue), that was threefold the initial value. This finding demonstrates that *S*. *bacillaris* strains can easily grow and develop in a wound on apples. Moreover, 1.3 x 10^6^ CFU/ml of grape must represent a suitable *S*. *bacillaris* inoculum level proposed in sequential fermentation to obtain wine with high glycerol concentration [27,30].

The fermentation performances in natural apple juice of the 14 non-*Saccharomyces* strains were tested, both in single-strain fermentation and in sequential fermentation, together with *S*. *cerevisiae*, to evaluate the possible positive effects on cider production. A cell concentration of 1.5×10^6^ cells/ml was used to inoculate apple juice, reproducing the concentration found on colonized apple wounds.

Single-strain fermentations in apple juice confirmed the *S*. *bacillaris* fructophilic character evidenced during grape fermentation, together with high glycerol production, high sugar residues and consequently low CO_2_ and ethanol production. All the sequential inocula allowed to complete fermentations, that lasted 7 days more than that of *S*. *cerevisiae* alone. The fermentation slowdown reported during sequential fermentations was previously evidenced in wine by Lemos et al. [30], and recently Englezos et al. [55] showed that yeast nitrogen requirement is not involved in *S*. *bacillaris*- *S*. *cerevisiae* interaction. Although sequential fermentation is slower than single-strain fermentation performed by *S*. *cerevisiae*, the overall time is still suitable for the industrial process needs. The fermentation slowdown during sequential fermentation seems to be a common feature, since it was also found when *Torulaspora delbrueckii* was used [56, 57].

In sequential fermentations, the presence of *S*. *bacillaris* strains significantly increased glycerol content in strains PAS13, PAS55, PAS92, PAS151, PAS173, FRI729, FRI754 and FRI7100, compared to the *S*. *cerevisiae* single-strain control. In winemaking, ethanol content and glycerol production positively contribute to palate fullness (“body”) of wine [58]. Therefore, high glycerol production is of great interest in cider, as ethanol level (8–9%) is generally lower than in wine. In all sequential fermentations, acetic acid concentration was lower than in *S*. *bacillaris* single-strain fermentations and comparable to that found in *S*. *cerevisiae* single-strain fermentation. Yeast acetic acid production is crucial, since this organic acid is the main responsible of volatile acidity that, if present at high level, confers an unpleasant vinegar aroma to the product. Finally, olfactory evaluation could not detect off-flavors related to volatile acidity and sulfur compounds.

In conclusion, this is the first study that demonstrates the ability of *S*. *bacillaris* to biologically control the apple blue mold caused by *P*. *expansum* without compromising product quality. The high wound colonization ability of *S*. *bacillaris* found in this work suggests that the use of this yeast as postharvest biocontrol agent on apple could positively influence the subsequent must fermentation, although the presence of *S*. *cerevisiae* is needed to complete the cider-making process. Finally, among the various strains tested, this work identified those that possess both biocontrol activity and technological properties. Further studies will be needed to optimize the protocol (cell concentration and treatments number) to assure both the efficacy of the selected strains as biocontrol agent during apple storage and the cider quality.

Our results provide a new approach to the application of non-*Saccharomyces* yeasts for apple juice fermentation, proposing a more integrated strategy for increasing cider quality.

## Supporting information

S1 Table Ability of the 14 *S*. *bacillaris* strains to reduce blue mold disease on apples. Lesion diameters (cm) measured on apple fruits in control wounds (inoculated with *P*. *expansum* only) and in inoculated wounds (treated with *S*. *bacillaris* 1 day before the inoculation with *P*. *expansum*). Lesion diameters were measured 7 days after *P*. *expansum* inoculation. During the experiment the apples were maintained at 25 °C, at high humidity.
(XLSX)

S2 Table Ability of *S*. *bacillaris* PAS13, PAS92, FRI29 and FRI7100 to reduce blue mold disease on apples. Lesion diameters (cm) measured on apple fruits in control wounds (inoculated only with *P*. *expansum*) and in inoculated wounds (treated with *S*. *bacillaris* 1 day before the inoculation with *P*. *expansum*). Lesion diameters were measured 7 days after *P*. *expansum* inoculation. During the experiment the apples were maintained at 25 °C, at high humidity.
(XLSX)
